# Fractures of the Inferior Maxilla

**Published:** 1860-10

**Authors:** W. A. Harris


### ARTICLE X.

*Fractures of the Inferior Maxilla.* Description of the gutta percha apparatus, designed for treatment of fractures of the inferior maxilla—Case exemplifying its good effects. By Dr. CORNE. Translated from the French by W. A. HARRIS, M. D., &c.

OBS. *Vertical fracture of the inferior maxilla—consecutive phlegmonous abscess—persistent displacement—employment of the gutta percha apparatus, on the thirteenth day—rapid cure.* Sauzet is a cuirassier in the 10th regiment, of a good constitution, born in the mountains of Drôme. On the thirtieth day of June he received a kick from a horse, in his back, which threw him forward; his head struck against the angle of a wall; for a few moments he was insensible. He was immediately carried to the hospital, where on the anterior part of the chest marks of contusion were discovered, but nothing in the dorsal region, which escaped the force of the hoof; pulse slow and depressed, sputa red and bloody, dullness of intellect, pain resulting from a violent contusion on the left angle of the maxilla, and under the mastoid process. There was no facial deformity, and auscultation of the chest revealed only a slight mucous râle.

The interior of the mouth was carefully examined, and I detected a little laceration of the gums, between the right canine and incisor teeth; and observed a fracture whose direction was upward, between the incisors and canine, and vertically downward; no contusion of the integument, no displacement; simple mobility with crepitation. The hemorrhage proceeded from the dental artery, but was not profuse.

I bound the neighboring teeth with a small waxed thread, and applied a bandage around the chin, directing continuous cold applications; in the evening there was headache, pulse accelerated, V. S. to about twelve ounces, cold applications. July 1st—dulness somewhat diminished, pulse good, no displacement, bloody sputa. Owing to the deficient intelligence and intractibility of the patient, this condition of things was of short duration, as he would not submit to the cold applications or bandage, and moreover was troubled with bronchial catarrh. He speaks, coughs, and spits without using any precautions.

2d. The linen thread was replaced by a metallic one, and the first chin bandage, by one padded and stiffened with gum. Diet, pectoral infusion, opiate potion.

3d. Last evening the patient had a chill; same swelling at the left angle of the jaw, with acute pains, bloody sputa, slight œdema of the face, and sub-maxillary engorgement. Milk, pectoral infusion and opiate potion.

4th. Vertical displacement, rupture of the metallic thread, febrile heat; mercurial frictions and emollient cataplasms under the maxilla.

5th. Sleeplessness, considerable vertical and antero-posterior displacement. The right fragment carried upwards and outwards by the action of the temporal, masseter and pterygoid muscles ; the left, drawn downwards by the platysma, omo, and genio-hyoid. After their reduction, I applied successfully the following apparatus, and the fragments preserved admirably their proper relations.

It is composed : 1st, of a buccal metallic gutter, parabolic in form, which is applied to the teeth near the fracture ; 2d, of a metallic plated chin piece, concave and parabolic ; 3d, two iron or steel bands, soldered to the buccal gutter, arranged to support the lower lip, and then descending vertically to be received in two screw rings, which are connected with the inferior plate. Two screws adapted to these bands, below the screw rings, admit of the approximation or separation, at will, of the two plates, whose action is identical with that performed by the fingers of the operator, during the reduction.

6th. Sanious sputa, difficulty in speaking and drinking, submaxillary pain and tumefaction ; the tumefaction and pain at the left angle of the maxilla have disappeared, œdema of the face continues. Milk, pectoral infusion, opiate lotion, laxative enema.

7th. During the night, in a violent paroxysm of coughing, the patient displaced the apparatus, and two of the incisors were luxated. Cephalalgia, sanious sputa, submaxillary tumefaction increased, with considerable displacement. Diet, pectoral infusion, cataplasms, mercurial inunctions, opiate potion.

8th. Punctures on a level with the submaxillary gland, escape of about an ounce and a quarter of laudable pus. Same dressing, omission of the apparatus.

9th. Fluctuation perceived between the hyoid bone and the chin, evacuation of about an ounce, by a second puncture. Sanious and purulent sputa, same displacement—simple dressing.

10th. Appearance good, facial œdema has disappeared, submaxillary phlegmon diminished, and furnishes but little pus ; the patient nourished with milk, soup and panada.

11th. General condition satisfactory; suppuration checked, but same displacement remains; dressing simple, milk and panada, pectoral infusion, opiate potion.

12th. Sleep, pulse regular, and appetite.

In view of the inefficiency of the retentive apparatus employed, after the persistent luxation of the two teeth, and the want of solidity of the others, and of the complication of a large submaxillary abscess, I decided to await a more complete improvement, intending, on the occurrence of this event, to act promptly in adjusting the fragments.

13th. Meanwhile there was published in the report of a meeting of the Chirurgical Society, an analogous case of fracture of the inferior maxilla, which M. Morel Lavallié had reduced and cured, by the employment of the gutta-percha; I made trial of his apparatus, and succeeded fortunately beyond my expectations.

I will not resume the consideration of the displacement, which, in relation to its height, was vertical, and to its thickness, antero-posteriorly. The reduction was made, and very easily executed. The left fragment is rendered motionless by the interposition of a piece of cork between the large molars; on this side, a small stick, about an inch long, and one-tenth of an inch thick, is introduced by separating the right labial commissure between the large molars; with this lever, whose point d'appui is at the extremity which bears upon the superior molars, whilst the power is at the other end, in the hand of the operator, by a simple depressing and rotating inward of the hand, we obtain the approximation of the resisting point, namely, the right fragment, which is in advance of that of the left side.

An assistant repeats the movement, and is charged with the coaptation. I then apply upon the dental arch, at the seat of fracture, a small band of gutta percha, parabolic in form, five centimeters long, twelve millimeters thick, previously softened in hot water; it is easy to mould it upon the teeth with the fingers, whilst the thumbs, placed under the inferior border of the maxilla, antagonize their action. Injections of cold water solidify this mould sufficiently, thereby enabling me to withdraw it without distortion; six teeth are found nicely indentated in their proper relation with the alveolar arch.

I cut and fashion this small cast before restoring it, and subsequently confine it with my first metallic apparatus. Nothing more complete: exactness of contention, solidity, freedom of functional duties, all are united; the face has resumed its proper form and normal appearance, the patient eats on the same day bread and farinaceous diet.

14th. Sleep good. I am careful that every thing is in place; as additional precaution, I apply as a support to the chin, a bandage crossed on the forehead, whose turns are carefully gummed. Soft bread and vegetables for diet.

15th. The bandage is solidified by the gum, the gutta percha cast remains in position, the patient speaks and eats freely.

16th to 31st. Appearance and general condition of the patient remain satisfactory, and he takes nourishment easily; two or three times during the night he has complained of pain at the seat of fracture. The submaxillary swelling decreases daily; care is taken to tighten a little the screws.

Aug. 1st. Removal of the apparatus—the gutta percha mould is perfect, without odor or deformity—the teeth are on a level—no displacement in their thickness—slight vertical projection at the seat of fracture, formed by the provisional callus. The luxated teeth have regained their relative position and solidity. By the opposing action of the fingers on the fragments, a little mobility is detected in consequence of the softness of the callus. The apparatus is cleaned and replaced.

10th. Ten days having passed without discomfort or suffering, the apparatus is again removed—the callus has become solid, and the tumor which it previously formed is flattened—apposition of the fragments perfect—a gummed bandage is substituted for the apparatus, which the patient retains as a prudential measure, till the 17th of August, the day of his leaving the hospital.

Numerous reflections present themselves on a review of this case ; let us enumerate the principal.

The absence of lesions at the seat of fracture, and the violent contusion which involved the left angle of the inferior maxilla, naturally induce us to believe that the fracture took place by contre-coup, doubtless from excess of curvature impressed upon the bone.

The accident, originally simple, was soon followed by formidable complications ; persistent displacement, submaxillary phlegmonous abscess, in which the fragments were bathed, difficulty of speech and deglutition, all of which are explained by the intractability of the patient, the vertical direction of the fracture, the opposing tractions which the strong muscles impress upon the fragments, and finally, by the constant motion of those which subserve the necessity of breathing, drinking and spitting.

It is not surprising to see the patience and care of the surgeon fail; I congratulate myself, after having experienced such complications, when others were also doubtless to be expected, in having triumphed over them, and I willingly yield to the desire of making public the success of my plan, so as to spare my fellow practitioners the difficulties which I have experienced.

There is nothing more simple than the reduction and coaptation, by making use of a small stick for a lever, while the impression is taken in gutta percha ; the dental mould is obtained without trouble, and with an exactness which is measured by the coaptation.

The construction of the plates and the metallic apparatus to complete the dressing, and give it firmness and solidity, offers other difficulties ; they may be judged of by the methods which are detailed in the books, apparatus, of Rudenick, of Busch, of Arnzelot, &c., all very imperfect, and whose efficacy is very conjectural. That which I

have employed requires an intelligent workman, but it is as simple in conception, as exact and convenient in application. After twenty-eight days application, the solidity of the callus permitted its removal, and the adjustment of the fragments left nothing to be desired; the two luxated incisors had resumed their position and solidity, ensheathed in the callus. No nervous symptoms were manifested, either in the direct track of the trifacial, or from sympathy with its principal branches; the general sensibility of the face, and special of the senses, also remained in their normal states.

NOTE.—Gramme, 15.438 grs. Troy. Centimetre, 0.39371 in. Millimetre, .03937 inch.

